# The Volatility of Data Space: Topology Oriented Sensitivity Analysis

**DOI:** 10.1371/journal.pone.0137591

**Published:** 2015-09-14

**Authors:** Jing Du, Arika Ligmann-Zielinska

**Affiliations:** 1 Department of Construction Science, Texas A&M University, Francis Hall 334, TAMU, College Station, TX 77840, United States of America; 2 Department of Geography, Michigan State University, 673 Auditorium Rd, Room 121, East Lansing, MI 48824, United States of America; Fondazione Edmund Mach, Research and Innovation Centre, ITALY

## Abstract

Despite the difference among specific methods, existing Sensitivity Analysis (SA) technologies are all value-based, that is, the uncertainties in the model input and output are quantified as changes of values. This paradigm provides only limited insight into the nature of models and the modeled systems. In addition to the value of data, a potentially richer information about the model lies in the topological difference between pre-model data space and post-model data space. This paper introduces an innovative SA method called Topology Oriented Sensitivity Analysis, which defines sensitivity as the volatility of data space. It extends SA into a deeper level that lies in the topology of data.

## Introduction

Sensitivity Analysis (SA) is “*the study of how uncertainty in the output of a model can be apportioned to different sources of uncertainty in the model input*” [1]. Although it is frequently perceived as an optional step in modeling that can be omitted without a significant loss of information, SA can play a critical role in scientific discovery [2]. It offers a variety of benefits to improve the relevance of modeling to science and technology, including[2–4]:

- identification of critical model factors by quantifying the contribution of each model input variable to the variability of its output, which later allows for efficient allocation of resources for data acquisition;
- legitimate model simplification, which is particularly important when investigating complex systems;
- contribution to theory development by discovering the most accurate representation of the modeled system;
- investigation of deep uncertainties in a variety of systems–a painful but necessary step of scientific discovery;
- comprehensive investigation of model behaviors to provide acceptable policy recommendations in scenario analysis, especially in the absence of outcome scenarios endorsed by stakeholders;
- information provision for stakeholders to develop a shared understanding about the studied problems.

Current SA technologies can be roughly classified into two groups: one-factor-at-a-time (OAT) and global SA. Despite the difference in evaluating the multidimensional model input space, the existing SA technologies are all value-based, where the quantified uncertainties in the model input and output are due to changes of input/output values. For example, a typical OAT examines the change of value of a particular model output when one of the inputs is altered in its value while all other inputs remain unchanged. Another example is a type of global SA based on model output variance decomposition, which results in sensitivity indices that reflect the fraction of output variance (in value) contributable to a certain input.

**Fig 1:**
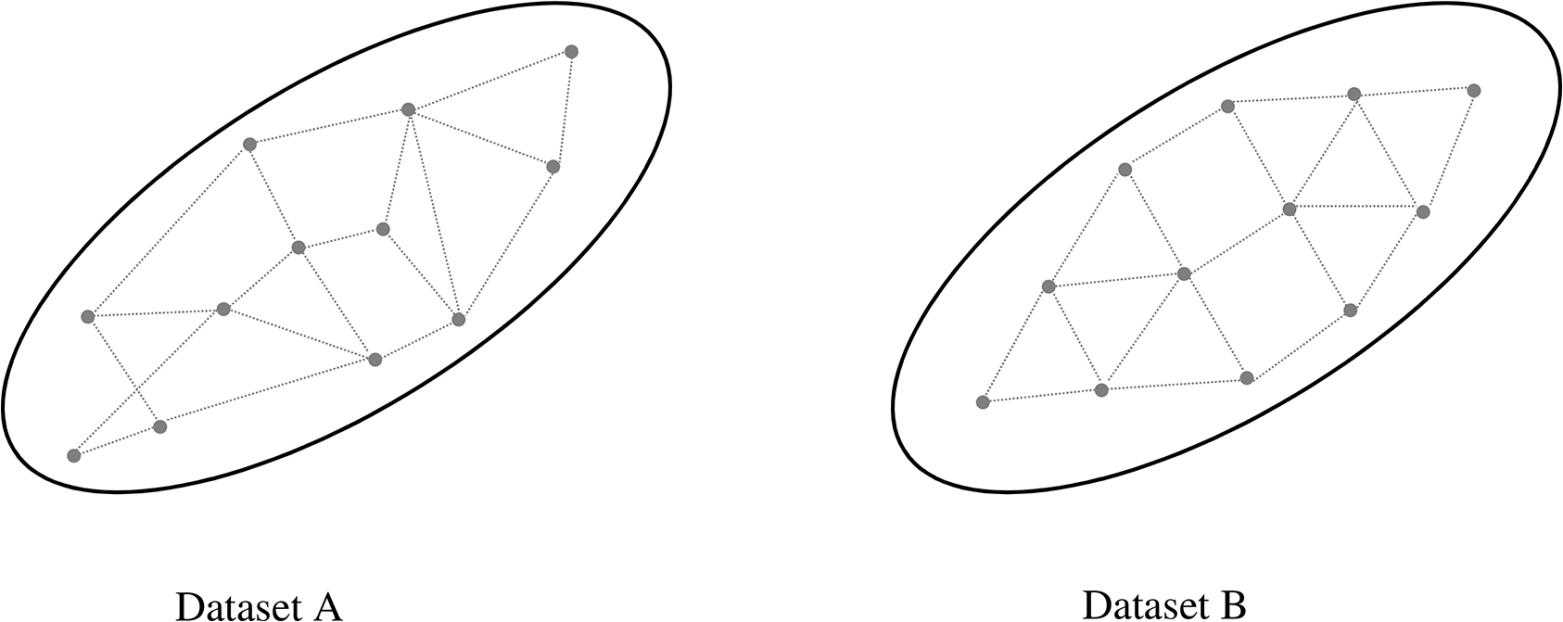
Two datasets with identical variance are different in nature.

This value-based paradigm of SA may result in limited insight into the nature of a given model. Fig 1 illustrates a situation when two datasets, A and B, have identical variance, but demonstrate very distinct topological nature of its data points. Specifically, dataset B has a more uniform spatial distribution while A shows a more random pattern. In scenario analysis, if each data point in datasets A and B represents an output scenario, value-based SA would provide little information about the relative "positions" of scenarios in the data space, and may lead to misleading conclusions about policies that satisfy a wide range of future conditions.

This study proposes a new approach to SA called Topology Oriented Sensitivity Analysis (TOSA). We postulate that model sensitivity comprises more than the variance of data, but also the volatility of data space. Consequently, not only does the proposed TOSA capture the change of value of model input and output, but it also provides the means of measuring topological changes of modeled data. It extends SA to a deeper level that lies in the topology of data. Following a brief introduction to the theoretical background, we give a complete description of TOSA. We then demonstrate the utility of TOSA using an agent-based model of shopping behavior as a case study.

## Background

### Sensitivity Analysis

Based on a comprehensive review of SA literature, Saltelli and Annoni (2010) state that most studies apply SA in an OAT fashion, i.e., changing the value of uncertain factors one-at-a-time while keeping the other factors constant [5]. It has already been found that OAT-SA is justified only for linear models [6, 7]. If the problem is nonlinear, OAT can lead to misleading conclusions [8, 9]. Failure to capture nonlinearities has also been found in regression and correlation based SA. Evidence indicates that regression based SA only works for linear models and its effectiveness depends on the goodness of fit [6]. Similarly, correlation measures are not effective at evaluating the sensitivity of complex models since nonlinearities are poorly taken into account in these measures [10].

To address the “curse” of nonlinearity, various remedies have been proposed including the method of Morris and the measure of importance. While the Morris method can account for nonlinearity, it assumes monotonicity, which does not always hold in complex models [10]. Moreover, the Morris method cannot differentiate between the effects caused by model nonlinearity and parameter interactions [10]. The importance measure is also of limited value because it only provides first-order effects (i.e., parameter interactions are not considered) and is very demanding computationally [6].

As a result, variance-based global SA (GSA) has recently received increased attention due to its model independence. In GSA, the unconditional variance of model output is decomposed into terms that account for individual factors plus terms that quantify the interactions among factors [11]. GSA has the capability to account for model nonlinearity and non-monotonicity, regardless of the generic assumptions of the underlying model [6, 10]. In a variety of studies, GSA has proven to do better than the more traditional SA approaches [12]. In the reported literature we identified the extended Fourier Amplitude Sensitivity Test (eFAST) and the method of Sobol as the most popular methods of performing GSA due to their proven track of performance. Sobol’s method will be used as a point of departure for the proposed TOSA. In order to explain TOSA, a brief discussion about the modeling theory is necessary.

### Redefine Models

**Fig 2:**
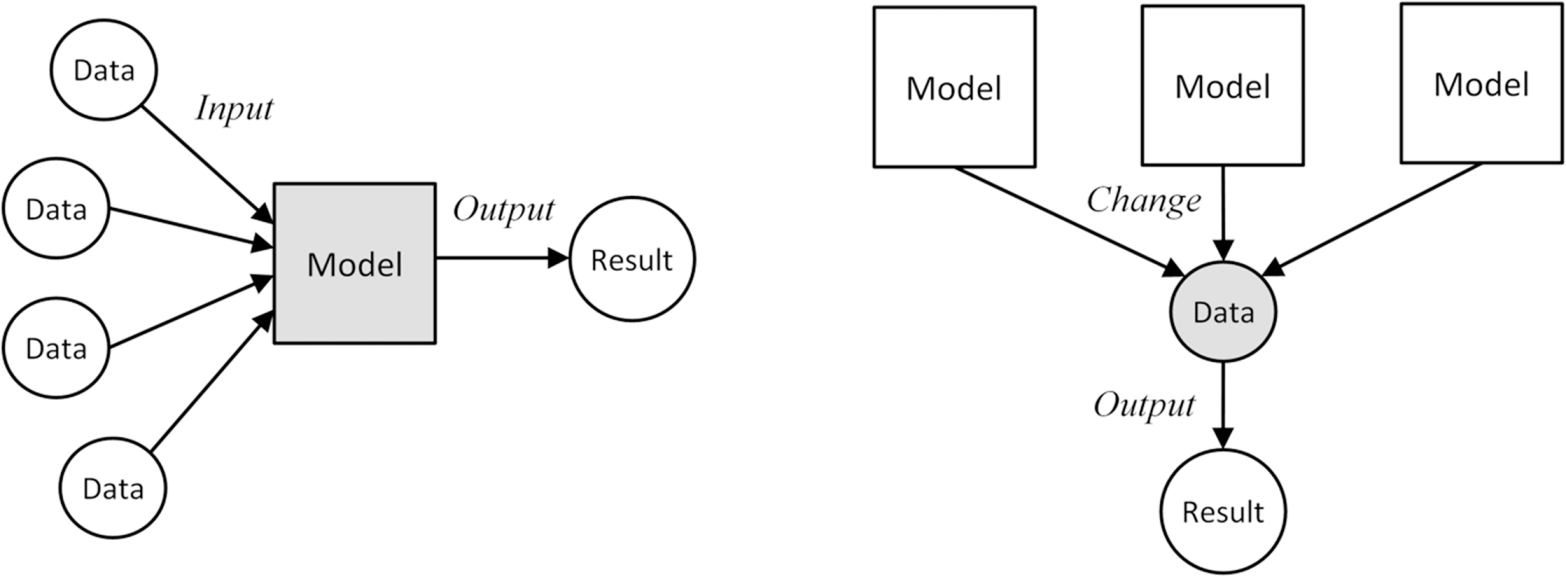
Model-Oriented simulation versus Data-Oriented simulation.

Miller and Page [13] have proposed a formal “model of models”, which addresses the underlying logic of system modeling. Roughly speaking, the “model of models” reflects the Input-Process-Output (IPO) view of modeling and simulation. The *input* consists of the present states of the system. The model *processes* the input data and generates an *output* in the form of new states of the system. This view puts the model at the center of the simulation process, as shown in Fig 2 left. In this “Model-Oriented” simulation, data is treated mainly as the input and output of a simulation process. The model comes first, then comes the data. An alternative way to look at the modeling process is the “Data-Oriented” simulation, where data occupies the center stage, and a variety of models are applied to it. Thus, under the “Data-Oriented” approach, the same data is changed by multiple models (Fig 2, right).

**Fig 3:**
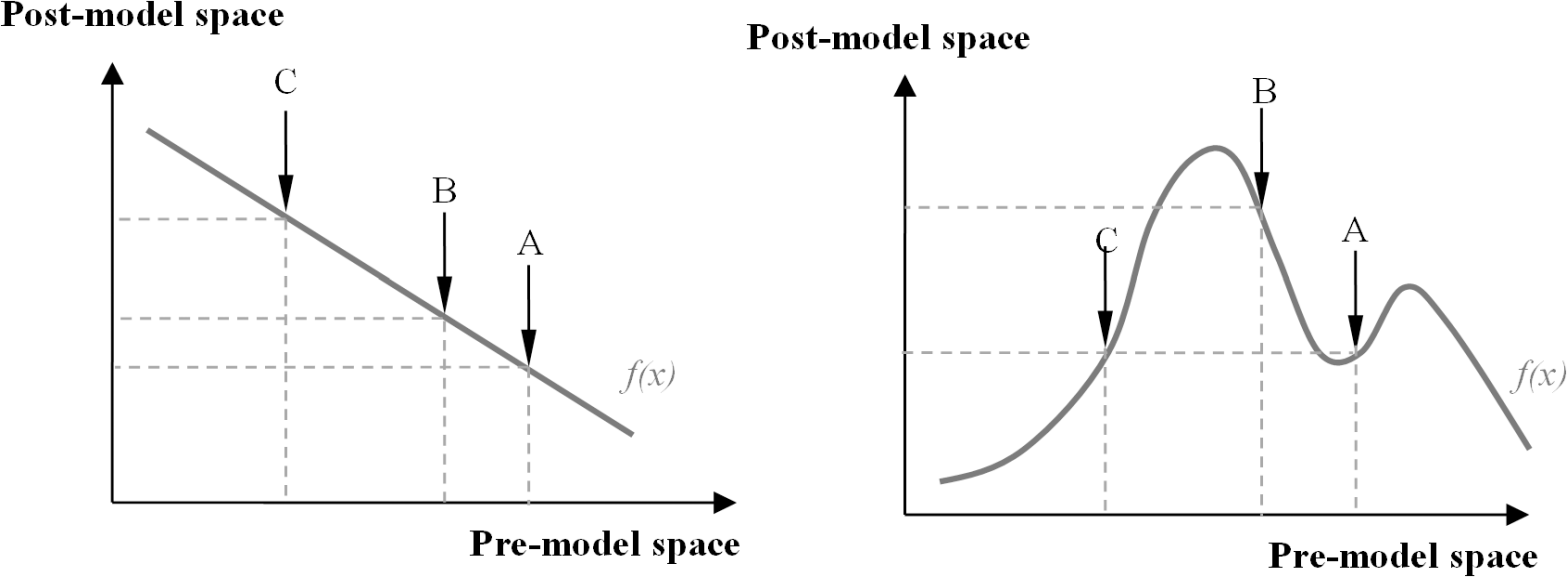
Model is a force that changes data space.

The “Data-Oriented” approach to simulation provides additional flexibility to define the modeled data. Data is represented as a space that can be changed by a model. Prior to the model, it is a multidimensional space that contains all the “known” information about the modeled system, After the model, it becomes a multidimensional space that contains all the “resulting states” of the system. A model is a force that changes the configuration of the data space. Fig 3 shows a simple example with one independent and one dependent variable. Assuming that the model is linear, the nearest neighbors A and B in the pre-model space (horizontal) remains the nearest neighbors in the post-model space (vertical; Fig 3 left). When the model is nonlinear, the A and B can end up farther away in the post-model space, with A and C becoming the nearest neighbors (Fig 3 right). Geometrically the space becomes distorted. We will use this data space distortion to redefine sensitivity.

**Fig 4:**
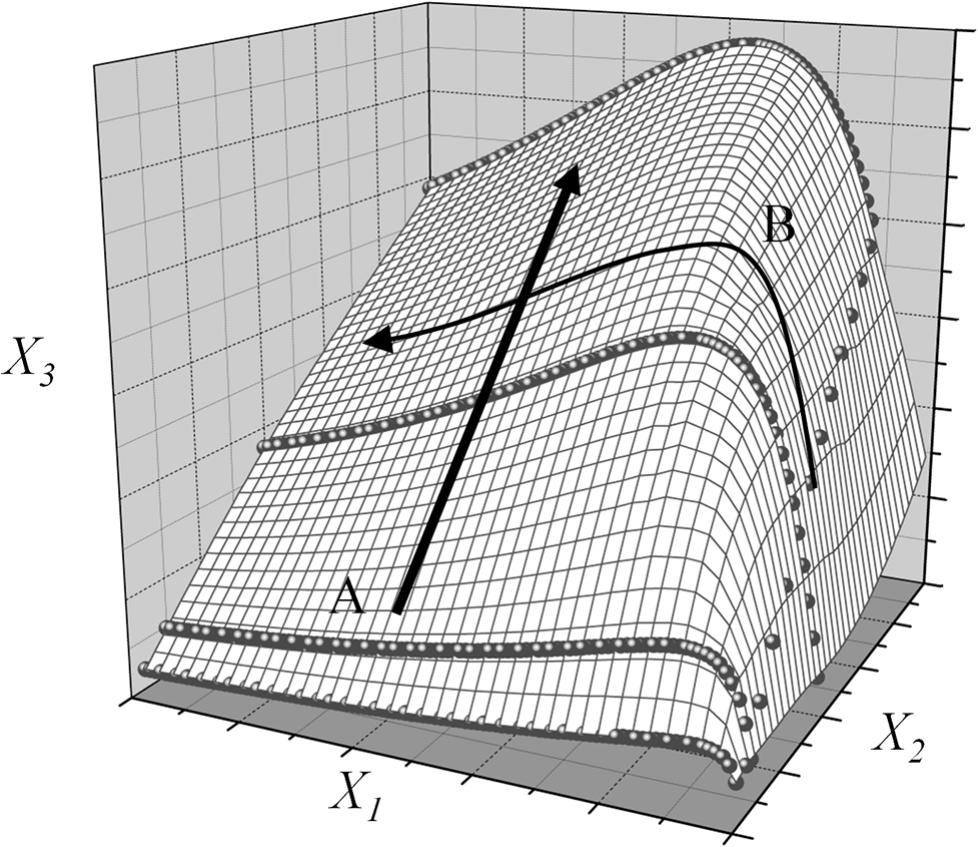
Heterogeneity of the data space.

Fig 4 illustrates a case of three dimensions. By traversing through the data space along track A, we capture less variability (uncertainty) than when traversing along track B. If we reduced the dimensionality from 3 to 2 by removing axis X_1_, we would not be able to reveal the actual space distortion. We can infer that X_1_ contains more information about the model and the data, and thus has a higher level of sensitivity i.e. in the example model outcome is more sensitive to X_1_ than X_2_ and X_3_.

Consequently, sensitivity of a particular variable can be defined as the absolute change of data space when the variable is removed from or added to the model. In the next section, we formulate TOSA to quantify the extent to which the data space distortion can be mitigated or augmented when a certain variable is removed or added.

## Topology Oriented Sensitivity Analysis (TOSA)

### Topological Measurements of Data Space

Define **X** = (**x**_1_, **x**_2_,…,**x**_n_)^T^ as the model input, where *n*∈[*1*, *∞*) is the number of inputs. For any given input *i* ∈ (*1*,*n*], there is a corresponding **x**_**i**_ = (x_i1_, x_i2_,…x_im_), where *m* is the number of input variables. Note that an input (*i*) and an input variable (**x**_**im**_) are different concepts, where the former one refers to a data point while the latter one refers to (one of many) variables defining the point. Similarly, define **Y** = (**y**_1_, **y**_2_,…, **y**_n_)^T^ as the output, where ***y***_***i***_ = (*y*_*i1*_, *y*_*i2*_,*…y*_*im’*_) and *m’* is the number of output variables. Let **Y** = f(**X**), where *f* is the model. It is therefore known that ∀**x**_i_∈**X**: **x**_i_∈ℝ^m^, where ℝ^m^ is a hyperspace with *m* dimensions and the *i* ^*th*^ input of the model is a data point in the hyperspace. Similarly, ∀**y**_i_∈**Y**: **y**_i_∈ℝ^m’^, where ℝ^m’^ is a hyperspace with *m’* dimensions, and the *i* ^*th*^ output is a data point (*y*_*i1*_, *y*_*i2*_,*…y*_*im’*_). Note that *m*≢*m’*, i.e., the number of input variables is not necessarily equal to the number of output variables; but it is assumed that the number of input points is equivalent to the number of output points (both are *n*). Finally, define **X**⊂ℝ^m^ as the input data space (i.e., pre-model data space) and **Y**⊂ℝ^m’^ as the output data space (i.e., post-model data space). Formula (1) shows that **X** is transitioned to **Y** through *f*.

$$\left(\begin{array}{cccc} x_{11} & x_{12} & \cdots & x_{1m}\\ x_{21} & x_{22} & \cdots & x_{2m}\\ \vdots & \cdots & \cdots & \vdots \\ x_{i1} & x_{i2} & \cdots & x_{im}\\ \vdots & \cdots & \cdots & \vdots \\ x_{n1} & x_{n2} & \cdots & x_{nm} \end{array}\right)\overset{f}{\to }\left(\begin{array}{cccc} y_{11} & y_{12} & \cdots & y_{1m\prime }\\ y_{21} & y_{22} & \cdots & y_{2m\prime }\\ \vdots & \cdots & \cdots & \vdots \\ y_{i1} & y_{i2} & \cdots & y_{im\prime }\\ \vdots & \cdots & \cdots & \vdots \\ y_{n1} & y_{n2} & \cdots & y_{nm\prime } \end{array}\right)$$ (1)

**Fig 5:**
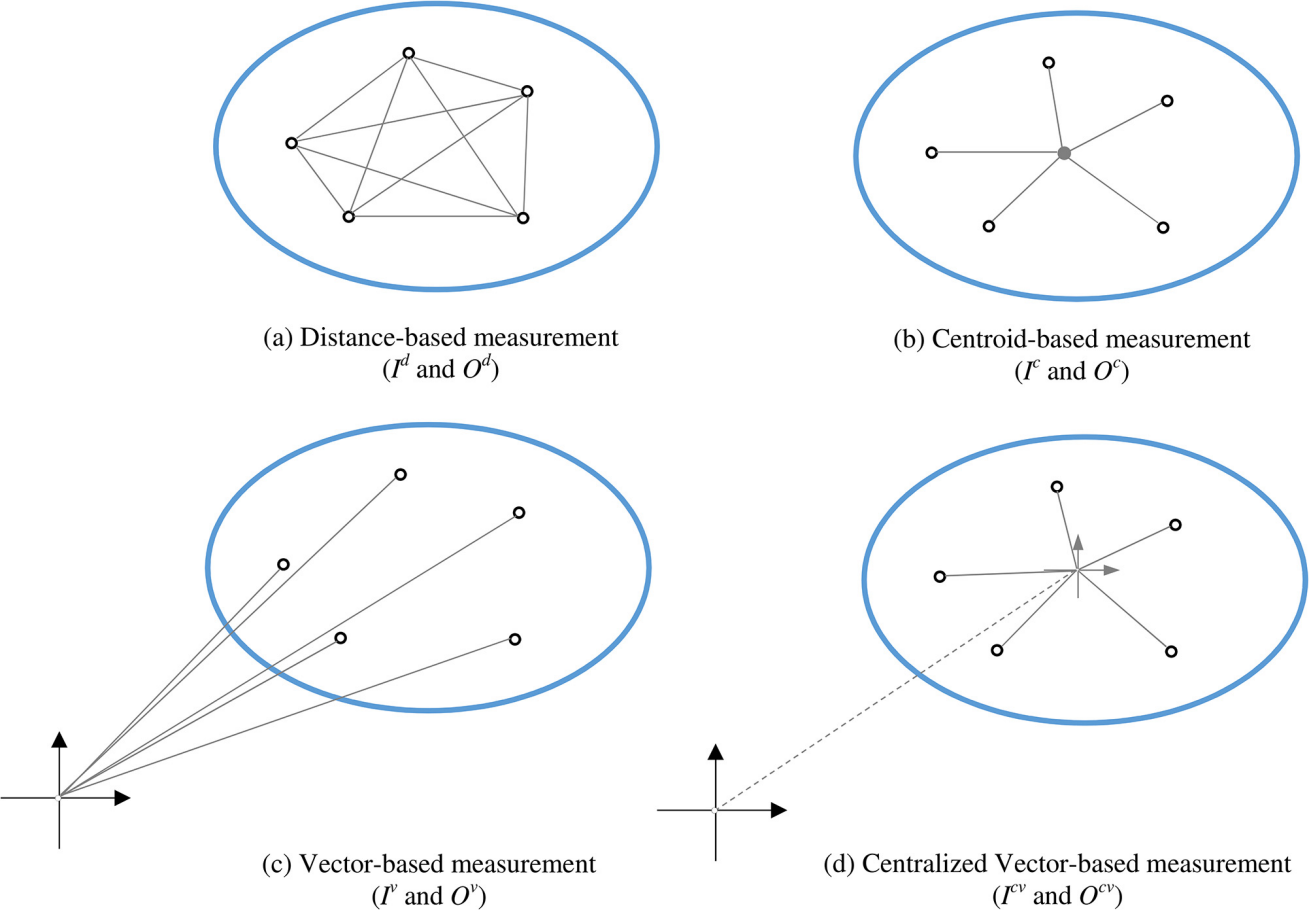
Four types of topological measurements of data space.

Where **X** is distorted by *f* and its topology is changed. The key step of the proposed SA is to measure the topology of input space (**X**) and output space (**Y**). We propose four ways of measuring the change in configuration: distance-based measurement, centroid-based measurement, vector-based measurement, and centralized vector-based measurement (Fig 5).

The distance-based measurement quantifies the distance between each pair of inputs, as illustrated in Fig 5 (A). Suppose the distance between a given pair of inputs **x**_i_ and **x**_j_ (or **y**_i_ and **y**_j_) is *D*_*ij*_, the average *D*_*ij*_ indicates the overall configuration of data space topology before (or after) the model takes effect. It is worth noting that **X** and **Y** are multidimensional spaces and therefore the Mahalanobis distance should be applied instead of the Euclidean distance [14]. Covariance *S* may exist among variables, exerting unnecessary influence on the calculation of Euclidean distance. The Mahalanobis distance standardizes the distance between any two data points by dividing Euclidean distance by *S* (covariance matrix), which better captures the relative locations of data points. Following the above notion, the distance between the data points *i* and *j* in the input space and output space are calculated as follows:

$$I_{ij}^{d}=\sqrt{{\left(x_{\text{i}}-x_{\text{j}}\right)}^{\text{T}}S_{X}^{-1}\left(x_{\text{i}}-x_{\text{j}}\right)}$$ (2)

$$O_{ij}^{d}=\sqrt{{\left(y_{\text{i}}-y_{\text{j}}\right)}^{\text{T}}S_{Y}^{-1}\left(y_{\text{i}}-y_{\text{j}}\right)}$$ (3)

And the distance-based measure for the input and output space topology is given by formulas (4) and (5), respectively:

$$I^{d}=\frac{2}{n\left(n-1\right)}\sum_{i=1}^{n-1}\sum_{j=i+1}^{n}I_{ij}^{d}$$ (4)

$$O^{d}=\frac{2}{n\left(n-1\right)}\sum_{i=1}^{n-1}\sum_{j=i+1}^{n}O_{ij}^{d}$$ (5)

Where *n* is the number of inputs (data points or cases) and *S*_*X*_ and *S*_*Y*_ are covariance matrices of input and output data, respectively. Distance-based measurement calculates *n(n-1)/2* pairs of data points. When *n* is big, the computation is nontrivial (e.g. for 100 data points, calculation amounts to 4950 pairs of points).

Data space configuration can also be measured as the distance of each data point to the centroid of the space, as illustrated in Fig 5B. Centroids of input and output spaces are given by:

$$x_{\text{c}}=\frac{1}{n}\times \sum_{i=1}^{n}x_{\text{i}},\;and\;y_{\text{c}}=\frac{1}{n}\times \sum_{i=1}^{n}y_{\text{i}}$$ (6)

For any given data point, if its distance to the centroid changes, then a distortion of the data space occurs. Using the Mahalanobis distance, the centroid-based measurement can be calculated as the average sum of squares of the distances to the centroid. Formulas (7) and (8) are used to calculate the distance between data point *i* and the centroid in the input and output space, respectively, and formulas (9) and (10) give centroid-based topological measurements of the input space and output space.

$$I_{ic}^{c}=\sqrt{{\left(x_{\text{i}}-x_{\text{c}}\right)}^{\text{T}}S_{\text{X}}^{-1}\left(x_{\text{i}}-x_{\text{c}}\right)}$$ (7)

$$O_{ic}^{c}=\sqrt{{\left(y_{\text{i}}-y_{\text{c}}\right)}^{\text{T}}S_{\text{Y}}^{-1}\left(y_{\text{i}}-y_{\text{c}}\right)}$$ (8)

$$I^{c}=\frac{1}{n}\sum_{i=1}^{n}I_{ic}^{c}$$ (9)

$$O^{c}=\frac{1}{n}\sum_{i=1}^{n}O_{ic}^{c}$$ (10)

Both the distance-based measurement and the centroid-based measurement use distance to quantify space topology. A common problem faced by both indices is the deterioration of the distance measure when the dimensionality of data is increasing. Beyer et al. [15] proved that the distance between any pair of data points starts to converge to an identical value when dimensionality reaches a certain point, as few as 10–15 dimensions. In other words, for any data point, with the increase in dimensionality, the distance to the nearest data point approaches the distance to the farthest data point:

$$lim_{d\to \infty }=\frac{dist_{max}-dist_{min}}{dist_{min}}=0$$ (11)

The above phenomenon means that, if the number of input variables (or output variables) is big enough, *I*_*ij*_ and *O*_*ij*_ will converge to an identical value. To overcome this limitation, a vector-based measurement is proposed.

As illustrated in Fig 5C, if the angle between two input data points **x**_i_ and **x**_j_ is *θ*_*ij*_, then $\text{cos}\left(\theta_{ij}\right)=\frac{x_{\text{i}}\cdot x_{\text{j}}}{\left\|x_{\text{i}}\|\|x_{\text{j}}\right\|}$. Note that *θ*_*ij*_ is the Euclidean angle. In order to eliminate the influence of covariance among variables, Mahalanobis angle *θ*_*ij*_^*M*^ is used [16]. By aggregating *cos(θ*_*ij*_^*M*^ *)*, the space topology can be quantified. Formulas (12) and (13) show the angles between any pair of data points *i* and *j* in the input space and output space. Formulas (14) and (15) give vector-based topological measurements of the input space and output space.

$$I_{ij}^{v}=\text{arccos}\left(\frac{x_{\text{i}}{}^{\text{T}}S_{\text{X}}^{-1}x_{\text{j}}}{\sqrt{x_{\text{i}}{}^{\text{T}}S_{\text{X}}^{-1}x_{\text{i}}}\sqrt{x_{\text{j}}{}^{\text{T}}S_{\text{X}}^{-1}x_{\text{j}}}}\right)$$ (12)

$$O_{ij}^{v}=\text{arccos}\left(\frac{y_{\text{i}}{}^{\text{T}}S_{\text{Y}}^{-1}y_{\text{j}}}{\sqrt{y_{\text{i}}{}^{\text{T}}S_{\text{Y}}^{-1}x_{\text{i}}}\sqrt{y_{\text{j}}{}^{\text{T}}S_{\text{Y}}^{-1}y_{\text{j}}}}\right)$$ (13)

$$I^{v}=\frac{2}{n\left(n-1\right)}\sum_{i=1}^{n-1}\sum_{j=i+1}^{n}I_{ij}^{v}$$ (14)

$$O^{v}=\frac{2}{n\left(n-1\right)}\sum_{i=1}^{n-1}\sum_{j=i+1}^{n}O_{ij}^{v}$$ (15)

It is worth noting that if data points are farther from the null vector (the origin of the coordinate), and the variance is very small, the angles between any two data points might be too small to demonstrate any significant change. In this case, data needs to be standardized to its center. Formulas (16) and (17) show the centralized angles between any pair of data points *i* and *j* in the input space and output space and formulas (18) and (19) give the centralized vector based topological measurements of the input space and output space.

$$I_{ij}^{cv}=\text{arccos}\left(\frac{\left(x_{\text{i}}-x_{\text{c}}{)}^{\text{T}}S_{\text{X}}^{-1}(x_{\text{j}}-x_{\text{c}}\right)}{\sqrt{{\left(x_{\text{i}}-x_{\text{c}}\right)}^{\text{T}}S_{\text{X}}^{-1}(x_{\text{i}}-x_{\text{c}})}\sqrt{{\left(x_{\text{j}}-x_{\text{c}}\right)}^{\text{T}}S_{\text{X}}^{-1}(x_{\text{j}}-x_{\text{c}})}}\right)$$ (16)

$$O_{ij}^{cv}=\text{arccos}\left(\frac{\left(y_{\text{i}}-y_{\text{c}}{)}^{\text{T}}S_{\text{Y}}^{-1}(y_{\text{j}}-y_{\text{c}}\right)}{\sqrt{{\left(y_{\text{i}}-y_{\text{c}}\right)}^{\text{T}}S_{\text{Y}}^{-1}(y_{\text{i}}-y_{\text{c}})}\sqrt{{\left(y_{\text{j}}-y_{\text{c}}\right)}^{\text{T}}S_{\text{Y}}^{-1}(y_{\text{j}}-y_{\text{c}})}}\right)$$ (17)

$$I^{cv}=\frac{2}{n\left(n-1\right)}\sum_{i=1}^{n-1}\sum_{j=i+1}^{n}I_{ij}^{cv}$$ (18)

$$O^{cv}=\frac{2}{n\left(n-1\right)}\sum_{i=1}^{n-1}\sum_{j=i+1}^{n}O_{ij}^{cv}$$ (19)

Given the failure of distance calculation in high-dimensional data, vector based measurements are recommended for complex multidimensional models.

### Topology Oriented Sensitivity Indices

For any given topological measurement, the change to the spatial relationship between the *i* ^*th*^ data point and the *j* ^*th*^ data point after the model takes effect (*T*_*ij*_) can be given by:

$$T_{ij}=\sqrt{{\left(O_{ij}-I_{ij}\right)}^{2}}$$ (20)

Where *I*_*ij*_ and *O*_*ij*_ represent any of the four proposed topological measurements for input space and output space, respectively. The topological change from the input data space to the output data space (*T*_*X*_) can be given by:

$$T_{X}^{c}=\frac{1}{n}\sqrt{\sum_{i=1}^{n}{\left(O_{ic}^{c}-I_{ic}^{c}\right)}^{2}}$$ (21)

$$T_{X}^{d,v,cv}=\frac{2}{n\left(n-1\right)}\sqrt{\sum_{i=1}^{n-1}\sum_{j=i+1}^{n}{\left(O_{ij}^{d,v,cv}-I_{ij}^{d,v,cv}\right)}^{2}}$$ (22)

Where *n* is the number of data points. *T*_*X*_ indicates the extent to which the input data space is distorted by the model given the full set of input variables, i.e., all variables from the *1* ^*st*^ to the *m* ^*th*^ are considered. On the other hand, we can define *T*_0_ as the topological change when none of the variables are considered. Trivially:

$$T_{0}=0$$ (23)

If removing a particular input variable, for example, the variable *i*, alters the value of *T*_*X*_, we can infer that the topological change from the input space to the output space is influenced by *i*. Let's denote *T*_*i*_ as the topological change after the variable *i* is added into the model, then:

$$T_{i}=T_{∼1,2,\ldots ,i-1,i+1,\ldots ,m}$$ (24)

where *T*_∼1,2,…,*i*−1,*i*+1,…,*m*_ is the topological change after all variables but variable *i* are removed from the model. Then total topological change of the data space *T(Y)* is given by:

$$T\left(Y\right)=\sum_{i=1}^{n_{i}}T_{i}+\sum_{i_{1}=1}^{n_{i_{1}}+n_{i_{2}}}\sum_{i_{2}=i_{1}+1}^{n_{i_{1}}+n_{i_{2}}}T_{i_{1}i_{2}}+\cdots +T_{X}$$ (25)

where *n*_*i*_ is the number of data points added into the model when variable *i* is added. *T(Y)* is the summation of topological changes when one variable, two variables … until all variables are added into the system. Following this notion, the Topology Oriented Sensitivity Index (TOSI) is given by:

$$TS_{i_{1},\ldots ,i_{k}}=\frac{T_{i_{1},\ldots ,i_{k}}}{T\left(Y\right)}$$ (26)

And the summation of all the TOSI equals 1:

$$\sum_{k=1}^{m}\sum_{i_{1}<..<i_{k}}^{m}TS_{i_{1},\ldots ,i_{k}}=1$$ (27)

If *k* = 1, then $TS_{i_{1},\ldots ,i_{k}}$ is called the Main Topology Oriented Sensitivity Index (MTOSI);

$$TS_{i}=\frac{T_{i}}{T\left(Y\right)}$$ (28)

if k ≥2, then $TS_{i_{1},\ldots ,i_{k}}$ is called the Interaction Topology Oriented Sensitivity Index (ITOSI). The Total Topology Oriented Sensitivity Index (TTOSI) is then defined as:

$$TS_{i}^{tot}=TS_{i}+TS_{i,∼i}=1-TS_{∼i}$$ (29)

Where *TS*_*i*, ∼*i*_ is the summation of all the $TS_{i_{1},\ldots ,i_{k}}$ that involve the variable *i* and at least one variable from (1,…, *i*-1, *i*+1, … *m*); and *TS*_∼*i*_ is the summation of all the *TS*_*i*, ∼*i*_ that do not involve any variable *i*. Consequently, $TS_{i}^{tot}$ represents the average topological change in the data space that is contributable to the input variable *i* through its sole influences and interactions with other variables.

### Calculation Procedure

TOSI computation requires a particular experimental design. This section explains the calculation steps. In order to make an easy demonstration, we use an example with three input variables *X*_*1*_, *X*_*2*_ and *X*_*3*_, and two output variables *Y*_*1*_ and *Y*_*2*._ The model is represented as *f*. Ten samples are generated in the simulation. Then we build a joint input-output matrix of five columns and ten rows:

$$\left(\begin{array}{ccccc} x_{11} & x_{12} & x_{13} & y_{11} & y_{12}\\ x_{21} & x_{22} & x_{23} & y_{21} & y_{22}\\ x_{31} & x_{32} & x_{33} & y_{31} & y_{32}\\ x_{41} & x_{42} & x_{43} & y_{41} & y_{42}\\ x_{51} & x_{52} & x_{53} & y_{51} & y_{52}\\ x_{61} & x_{62} & x_{63} & y_{61} & y_{62}\\ x_{71} & x_{72} & x_{73} & y_{71} & y_{72}\\ x_{81} & x_{82} & x_{83} & y_{81} & y_{82}\\ x_{91} & x_{92} & x_{93} & y_{91} & y_{92}\\ x_{101} & x_{102} & x_{103} & y_{101} & y_{102} \end{array}\right)$$ (30)

- Step 1: Generate a list of (*n*) input vectors (1*m vectors) using a random number sampling approach. *m* is the number of input variables and *n* is the number of generated samples. In our example, there are three input variables *X*_*1*_, *X*_*2*_ and *X*_*3*_, and ten randomly generated samples. The input values are showed in the following 3*10 matrix, where the elements are the randomly generated numbers.

$$\left(\begin{array}{ccc} x_{11} & x_{12} & x_{13}\\ x_{21} & x_{22} & x_{23}\\ \vdots & \ddots & \vdots \\ x_{101} & x_{102} & x_{103} \end{array}\right)$$ (31)

- Step 2: Execute the model *n* times with the generated input vectors. Each input vector is a line in the above matrix. In our example, for the first vector (*x*_*11*_, *x*_*12*_, *x*_*13*_), we obtain (*y*_*11*_, *y*_*12*_ *) = f*(*x*_*11*_, *x*_*12*_, *x*_*13*_). This step is repeated until the last execution (*y*_*101*_, *y*_*102*_ *) = f*(*x*_*101*_, *x*_*102*_, *x*_*103*_).
- Step 3: Calculate *T*_*X*_ following formulas (21) and (22). Matrix (30) is used to calculate *T*_*X*_. Note there are four types of *T*_*X*_ specified by equations (2) through (19).
- Step 4: Calculate the average value of the *n* samples given any input variable *X*_*i*_, denoted as $\bar{x_{i}}$, and remove input vectors except $\left(x_{1},x_{2},\ldots \;,\hat{x_{i}},\ldots \;,x_{m}\right)$, where $\hat{x_{i}}\approx \bar{x_{i}}$. Similarly, remove output vectors except $\left(y_{1},y_{2},\ldots \;,\hat{y_{i}},\ldots \;,y_{m\prime }\right)$, where $\left(x_{1},x_{2},\ldots \;,\hat{x_{i}},\ldots \;,x_{m}\right)\overset{f}{\to }\left(y_{1},y_{2},\ldots \;,\hat{y_{i}},\ldots \;,y_{m\prime }\right)$. For example, we calculate the average value of all samples given *X*_*1*_. Suppose the average value is close to *x*_*21*_, *x*_*51*_ and *x*_*71*_, then we remove all input vectors and corresponding output vectors other than the second, the fifth and the seventh vectors. By doing it, the randomness of *X*_*1*_ has been ruled out (only leaving the average value), and thus its impact on the outputs is eliminated. The new matrix is:

$$\left(\begin{array}{ccccc} x_{21} & x_{22} & x_{23} & y_{21} & y_{22}\\ x_{51} & x_{52} & x_{53} & y_{51} & y_{52}\\ x_{71} & x_{72} & x_{73} & y_{71} & y_{72} \end{array}\right)$$ (32)

- Step 5: Calculate *T*_1,2,…,*i*−1,*i*+1,…,*m*_ = *T*_∼*i*_ following formulas (21) and (22). In our example, matrix (32) is used to calculate the new *T*_*X*_. The results is denoted as *T*_∼1_ to illustrate that *X*_*1*_ has been removed. After the calculation of this step is complete, the removed vectors are put back to matrix (32).
- Step 6: Repeat steps 4 and 5 until all *x*_*i*_ are consecutively removed, and the corresponding *T*_*~i*_’s are calculated. In our example, *T*_∼1_, *T*_∼2_ and *T*_∼3_ are calculated, which means *X*_*1*_, *X*_*2*_ and *X*_*3*_ are removed separately from matrix (30).
- Step 7: For any input vector $\left(x_{1},x_{2},\ldots \;,\hat{x_{i}},\ldots \;,x_{m}\right)$, calculate the average value of input variable *x*_*j*_ denoted as $\bar{x_{j}}$, where *i*≠*j*, and remove input vectors except $\left(x_{1},x_{2},\ldots \;,\hat{x_{i}},\ldots \;,\hat{x_{j}},\ldots \;,x_{m}\right)$, where $\hat{x_{i}}\approx \bar{x_{i}}$ and $\hat{x_{j}}\approx \bar{x_{j}}$. Similarly, remove output vectors except $\left(y_{1},y_{2},\ldots \;,\hat{y_{i}},\ldots \;,\hat{y_{j}},\ldots \;,y_{m\prime }\right)$, where $\left(x_{1},x_{2},\ldots \;,\hat{x_{i}},\ldots \;,\hat{x_{j}},\ldots \;,x_{m}\right)\overset{f}{\to }\left(y_{1},y_{2},\ldots \;,\hat{y_{i}},\ldots \;,\hat{y_{j}},\ldots \;,y_{m\prime }\right)$. In our example, suppose *X*_*1*_ has been removed (matrix (32)). Then the average value of the remaining samples given *X*_*2*_ is calculated, which is close to *x*_*22*_ and *x*_*72*_. Thus we need to remove the fifth input vector and the corresponding output vector. The new matrix is:

$$\left(\begin{array}{ccccc} x_{21} & x_{22} & x_{23} & y_{21} & y_{22}\\ x_{71} & x_{72} & x_{73} & y_{71} & y_{72} \end{array}\right)$$ (33)

Where the impact of both X1 and X2 on outputs have been eliminated. It is therefore possible to evaluate the importance of these input variables by “removing” them from the model.

- Step 8: Calculate *T*_1,2,…,*i*−1,*i*+1,…,*j*−1,…,*j*+1,…,*m*_ = *T*_∼*ij*_ following formulas (21) and (22). In our case, matrix (33) is used to calculate the new *T*_*X*_, which is denoted as *T*_∼12_, to illustrate that *X*_*1*_ and *X*_*2*_ have been removed.
- Step 9: Repeat steps 7 and 8 until *x*_*1*_,…,*x*_*k*_ (2≤*k*≤*m*) are removed, and all the corresponding *T*_*~1*,*2*,*…*,*k*_ (2≤*k*≤*m*) are calculated. In our case, the calculated *T*_*X*_’s include *T*_∼1_,*T*_∼2_,*T*_∼3_,*T*_∼12_,*T*_∼13_ and *T*_∼23_.
- Step 10: Calculate MTOSI, ITOSI and TTOSI following formulas (25) through (29).

## Case Study

### The URBAN model

**Fig 6:**
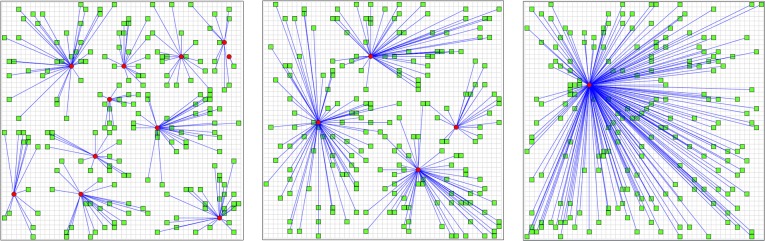
The URBAN model—sample results.

We demonstrate the proposed TOSA using a model called URBAN, an agent-based model (ABM) developed to investigate how individual shopping travel behaviors reshape urban configuration [17]. Two types of agents are modeled in URBAN: *Store* and *Person*. As illustrated in Fig 6, the red dots represent stores, and the green squares are people. When an individual decides to go shopping at a particular store, a connection is established (represented by a blue line in Fig 6). The go/no go decision of a person depends upon three factors: (1) the utility of a store (including the size of the store, the selection of goods, etc.); (2) store accessibility (including store distance and traveling expenses); and (3) the socioeconomic status of the individual. If a store is visited by more people, it maintains higher profits and grows in size, which in turn becomes more attractive to other potential shoppers; in contrast, if a store is visited less frequently, it starts to lose profit, shrinks in size, and ultimately runs out of business (it is removed from the ABM).

Table 1 summarizes the main input and output variables of URBAN. Note that, for the demonstration purposes, the initial number of stores, the initial number of people, and the size of urban space are held fixed in the computation of TOSI’s. Four critical input variables are examined in the simulations: preference for store utility (*α*), preference for accessibility (*β*), dollar amount of purchase, and store operating cost per day. Note that *α* and *β* are directly related to individual decision making: if the value of *α* is bigger for an agent, it means she/he cares more about the store utility; and if *β* is bigger, it means that accessibility is more important to the agent. The other two input variables, *dollar amount of purchase* and *store operating cost per day*, influence the dynamics of the store status: if a store has a higher daily operating cost (in percent of the cash reserve), it may run out of business faster if not enough income is obtained. On the other hand, if dollar amount of purchase is higher, the stores may be able to maintain the income. The output variables of interest are the final number of stores, the total walking distance and the total driving distance. These output variables are used to determine the final urban configuration.

**Table 1:** Input and output variables of the URBAN model.

Symbol	Variable name	Values	Symbol	Variable name	Values
	***Input***		*X6*	Initial # of stores	20
*X1*	Preference for store	0~6, step 0.25	*X7*	Initial # of people	200
*X2*	Preference for travel	0~6, step 0.25		***Output***	
*X3*	$ amount of purchase	N (100, 20)	*Y1*	Final number of stores	N/A
*X4*	Store operating cost per day	N (0.01,0.001)	*Y2*	Total walking distance	N/A
*X5*	Urban space size	50*50 gird where a grid cell equals 100^2^ square feet	*Y3*	Total driving distance	N/A

The dollar amount of purchase and the store operating cost per day were assumed to follow a normal distribution (Table 1); socioeconomic status, measured as household income, was assumed to follow a Pareto distribution.

### TOSI

All the four types of TOSI’s were calculated, including the distance-based indices, the centroid-based indices, the vector-based indices, and the centralized vector based indices. The *a* and *β* coefficients were changed in a OAT (one at a time) manner from 0 to 6, at the step 0.25. Because Probability Density Functions (PDFs) were used to describe certain input variables, for a given combination of *a* and *β*, the simulation was repeated 30 times to generate a statistically sound results. As a result, a total of 18,750 simulations were performed. The input data space is therefore a seven-dimensional space with 18,750 data points, and the output space is a three-dimensional space with the same number of data points.

The centroid based indices were first calculated. Fifteen terms were calculated including all the main and interaction topology-oriented indices, each involving 18,750 matrix calculations. Given the difficulty of calculation, a Visual Basic (VB) program was developed to facilitate the calculation. The selection of VB is a practical choice. Many business practices rely on Microsoft Excel. It would be easier for nonacademic users to apply our tools in Excel as a VB application (VBA). For academic users, we also provided the tools programed in SAS and R [18], which more efficiently handle matrix data structures.

Table 2 summarizes the results. After normalizing the result to 100% ($\%\;TS_{i}\;and\;\%\;TS_{i}^{tot}$), we identified the *preference for the store utility* (X1) and *preference for accessibility* (X2) to be more influential on the topological change than the *dollar amount of purchase* (X3) and *store operating cost* (X4).

**Table 2:** Centroid based TOSI’s.

*No*.	*Combination*			
*1*	*X1*	*X2*	*X3*	*X4*
*2*		*X2*	*X3*	*X4*
*3*	*X1*		*X3*	*X4*
*4*	*X1*	*X2*		*X4*
*5*	*X1*	*X2*	*X3*	
*6*			*X3*	*X4*
*7*		*X2*		*X4*
*8*		*X2*	*X3*	
*9*	*X1*			*X4*
*10*	*X1*		*X3*	
*11*	*X1*	*X2*		
*12*	*X1*			
*13*		*X2*		
*14*			*X3*	
*15*				*X4*
	*X1*	*X2*	*X3*	*X4*
*T*_*i*,∼i_	*0*.*3396*	*0*.*3318*	*0*.*2283*	*0*.*2310*
*TS*_*i*_	*0*.*2239*	*0*.*2211*	*0*.*0936*	*0*.*0943*
$TS_{i}^{tot}$	*0*.*4427*	*0*.*4326*	*0*.*2977*	*0*.*3012*
% *TS*_*i*_	*35*.*4%*	*34*.*9%*	*14*.*8%*	*14*.*9%*
$\%\;TS_{i}^{tot}$	*30*.*0%*	*29*.*3%*	*20*.*2%*	*20*.*4%*

X1: preference for the store utility (α)

X2: preference for accessibility (β)

X3: dollar amount of purchase

X4: store operating cost per day.

We also calculated the other three types of TOSI. Compared to the centroid-based indices, the calculation was more difficult, as each of the 15 terms involves 175,771,875 matrix calculations. A set of VB programs was developed to facilitate the calculation. The results are summarized in Table 3. Although different in exact values, all TOSI’s suggest stronger influences of *preference for the store utility* (X1) and *preference for accessibility* (X2).

**Table 3:** Calculation results.

*MTOSI*	*X1*	*X2*	*X3*	*X4*
*Distance based*	*28*.*9%*	*28*.*1%*	*21*.*2%*	*21*.*8%*
*Centroid based*	*35*.*4%*	*34*.*9%*	*14*.*8%*	*14*.*9%*
*Vector based*	*35*.*0%*	*31*.*8%*	*18*.*9%*	*14*.*3%*
*Centralized Vector based*	*29*.*6%*	*29*.*4%*	*20*.*6%*	*20*.*4%*
***TTOSI***	***X1***	***X2***	***X3***	***X4***
*Distance based*	*28*.*3%*	*27*.*8%*	*21*.*7%*	*22*.*2%*
*Centroid based*	*30*.*0%*	*29*.*3%*	*20*.*2%*	*20*.*4%*
*Vector based*	*31*.*3%*	*29*.*9%*	*21*.*5%*	*17*.*3%*
*Centralized Vector based*	*28*.*7%*	*28*.*7%*	*21*.*3%*	*21*.*4%*

**Fig 7:**
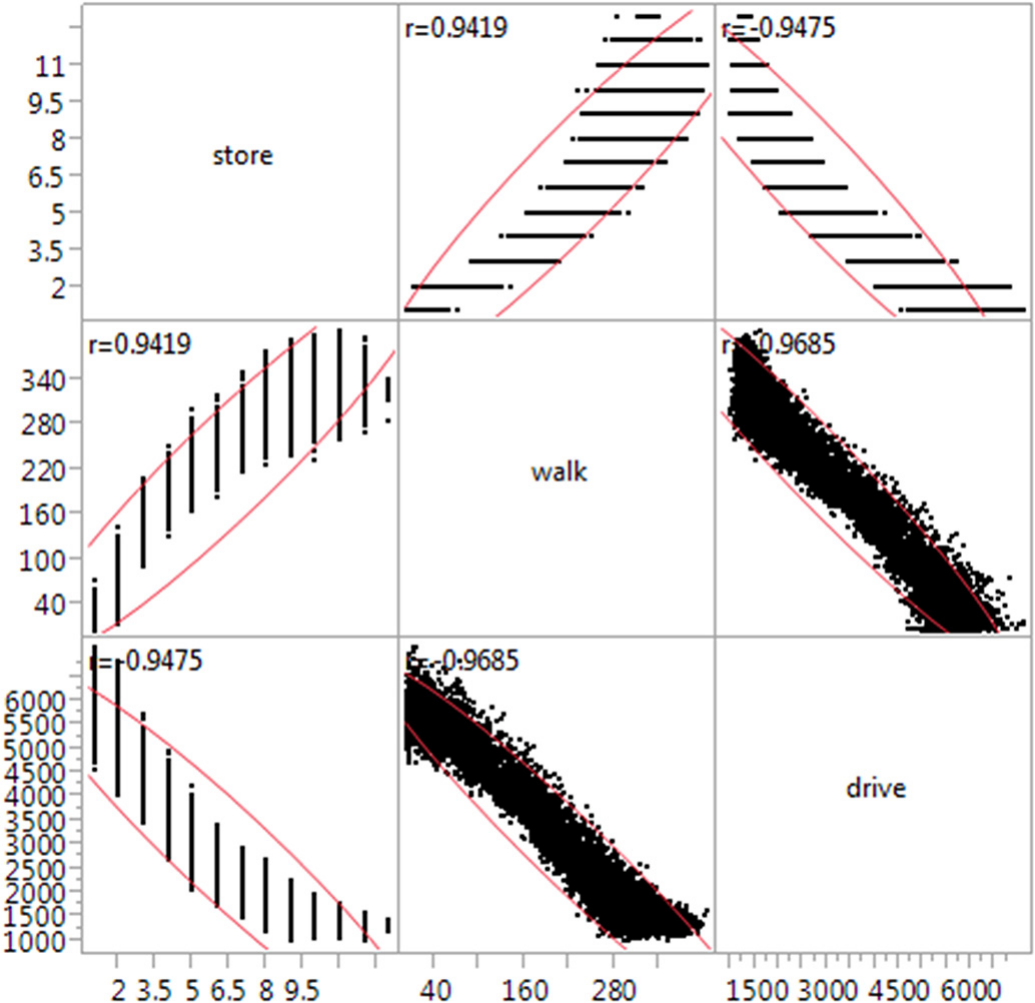
Correlation of the three output variables.

In order to evaluate the difference between TOSA and traditional SA, we performed an OAT SA on the same data. To be noted, unlike TOSA that gives a single sensitivity index in spite of the number of output variables, the OAT SA needs to be done for each of the output variables. Fig 7 shows that the three output variables—final number of stores (store), the total walking distance (walk) and the total driving distance (drive)—are highly correlated. As a result, we only present the results of OAT SA for output “drive”.

**Fig 8:**
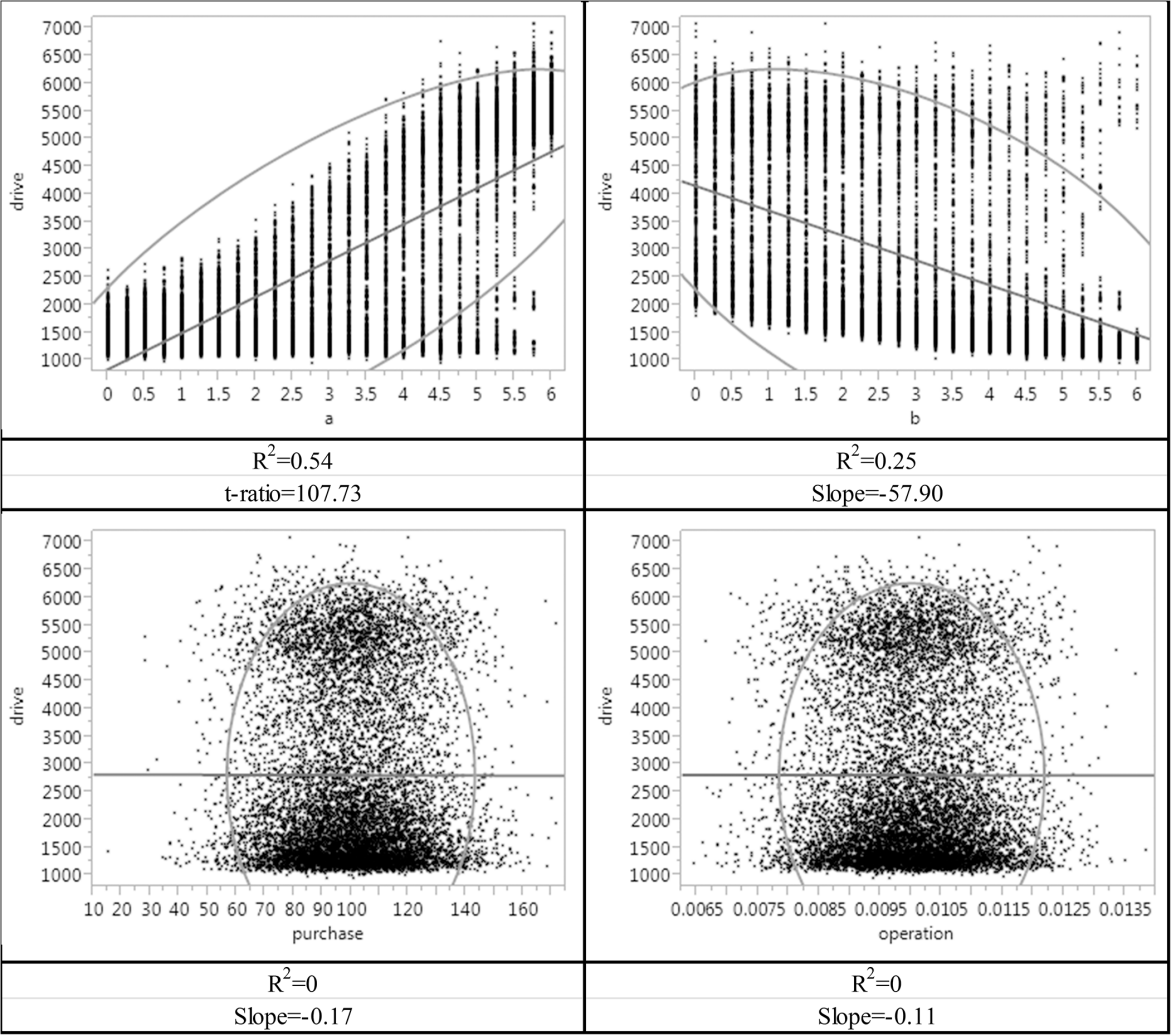
OAT sensitivity analysis on total driving distance.

To obtain comparable measures, we summarized the results of OAT using linear regression. As shown in Fig 8, coefficients *α* and *β* are more influential on the output variable “drive”, while “purchase” and “operation” do not show any observable impact on this output. If using the t-ratio of the linear regression (which is the standardized coefficient of input variable in a regression analysis) to evaluate the degree of impacts, *α* outperforms *β*. In other words, according to the OAT SA, *preference for the store utility* (X1) and *preference for accessibility* (X2) can significantly affect the total driving distance, with the former exhibiting stronger influence. However, the OAT SA does not support the impact of *dollar amount of purchase* (X3) or *store operating cost* (X4) on the total driving distance.

**Fig 9:**
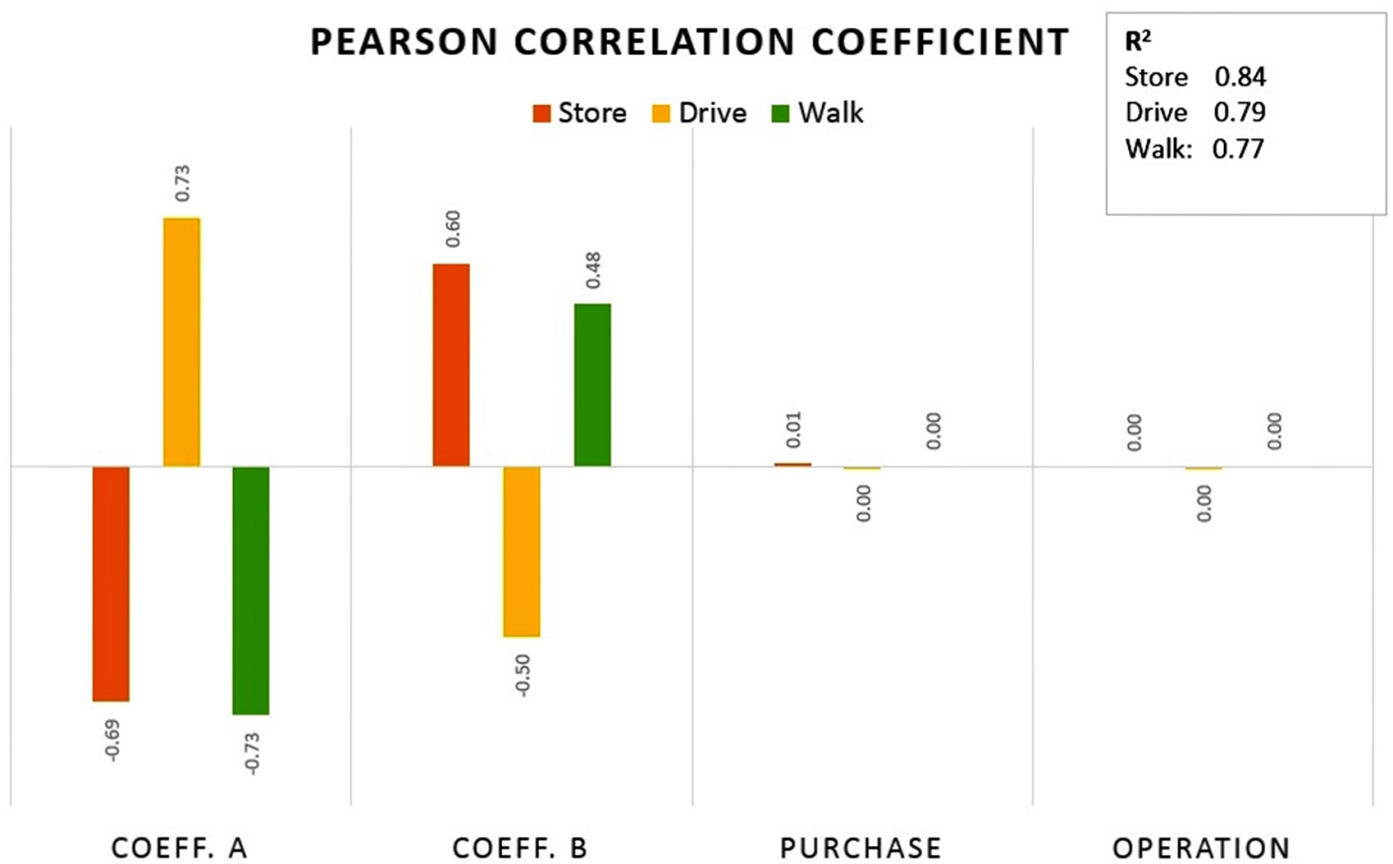
Pearson correlation coefficient calculated for the three output variables.

We also used Pearson’s correlation coefficient (r)—one of the simplest measures of global SA [19]. Fig 9 depicts the results of the analysis, together with the coefficients of determination. The following observations can be made. Given all three output measures, the model behaves fairly linearly as about 80% of the influence can be attributed to individual inputs (R^2^ ~ 0.8). The influence of inputs is similar for “store” and “walk” with the coefficients *α* and *β* mostly affecting the variability of the outputs. Note that where the impact of *α* is negative, the impact of *β* is positive. For ‘drive’, the impact is essentially inverted. In absolute terms, however, *α* and *β* coefficients are the most influential on all three output variables, with *α* slightly dominating *β*.

**Fig 10:**
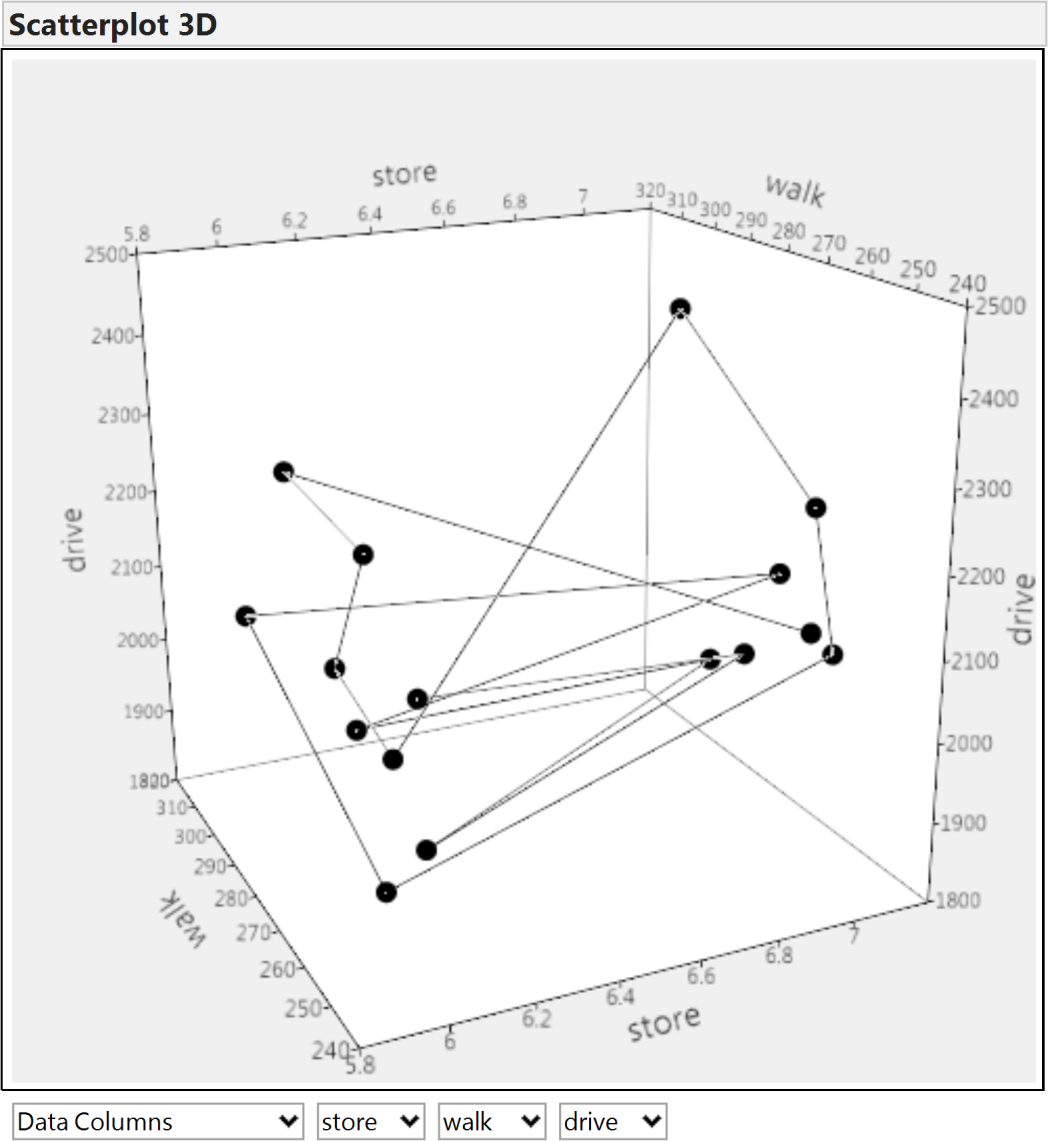
The volatile outcome when the values of X1 and X2 are fixed to 3.0.

After comparing the results of the three methods, we found that TOSA, OAT SA and Global SA (based on the correlation coefficient), can lead to quite different conclusions. Although all three methods unanimously suggest that *reference for the store utility* (X1) is the most influential factor and *preference for accessibility* (X2) is the second, TOSA also indicates the importance of *dollar amount of purchase* (X3) or *store operating cost* (X4). It is because the existence of *dollar amount of purchase* (X3) or *store operating cost* (X4) can affect the level of topological change of the data, which is the foundation of TOSA calculation. Practically, it means that although altering *dollar amount of purchase* (X3) or *store operating cost* (X4) will not change the expected number of stores, or the expected walk/driving distance, the specific combination of the three urban setup indicators has actually changed. For example, Fig 10 illustrates the simulation result when *dollar amount of purchase* (X3) and *store operating cost* (X4) are changing, while the values of *reference for the store utility* (X1) and *preference for accessibility* (X2) are both fixed to 3.0. As shown, although the expected outcome is always the same (it is the centroid of the 3D scatter plot), it actually represents very different futures–without changing X1 and X2, the maximum driving distance is still 22.5% longer than the minimum driving distance in only 16 simulations. The output data space is still very volatile, suggesting potentially distinct futures. Traditional value-based SA methods fail to capture this nuance because this piece of subtle information is hidden behind data topology rather than the mean value or its variance. Clearly, TOSA provides a different yet complementary angle to interpret model sensitivity.

A further comparison has highlighted the differences between TOSA and traditional SA. Traditional value-based SA methods rely heavily on summary statistics (variance, mean) which may fail when dealing with non-monotonic and non-additive models [20, 21]. While useful in many circumstances, the correlation coefficient (and its derivatives like Spearman, standardized regression coefficients etc.) is derived from a linear model, and does not provide any information on the interaction effects among inputs which, in our example, account for about 20% of model variability. Published research suggests that some of the inputs may not be influential singly, but may substantially affect the variability of results when evaluated in combination with other inputs, contributing to higher-order effects [22]. In addition, the measures of sensitivity may be different for different output variables (in our case, for each input variable, there is a separate sensitivity index for “store”, “walk” and “drive” respectively), posing a challenge to a modeler when deciding on factor fixing and model simplification. As an alternative, we could use ANOVA-like measures of sensitivity (aka variance-based global SA), but these methods require a quasi-random experimental design, which may be inappropriate when other types of post-processing analyses (based on parametric statistics) are also employed to the output data.

## Discussion

Model sensitivity has to be evaluated in relation to the specific context of a modeling study. In general however, the concept of model sensitivity relates to the relationships between model input and output uncertainties. Model SA should therefore measure the degree of change after the model takes effect. Most SA methods define uncertainty using statistical denotations, either as the absolute change in values, or as their variance. We argue that topology, which is not present in the commonly-utilized approaches to SA, may provide additional useful information on model behavior and the complexity of relationships between its inputs and outputs.

The proposed TOSA attempts to quantify the topological difference between model input data space and its output data space. It builds on a view of data-oriented simulation (Fig 2), in which models are external mechanisms that distort the data space. A cross-paradigm property of models is their ability to alter the data space topology. In this light, sensitivity is an indicator of the volatility of data space: if adding a dimension (an input variable) strengthens the volatility of the modeled data space, the outcome is sensitive to the added dimension. This new angle of SA is a promising avenue for model exploration and evaluation.

**Fig 11:**
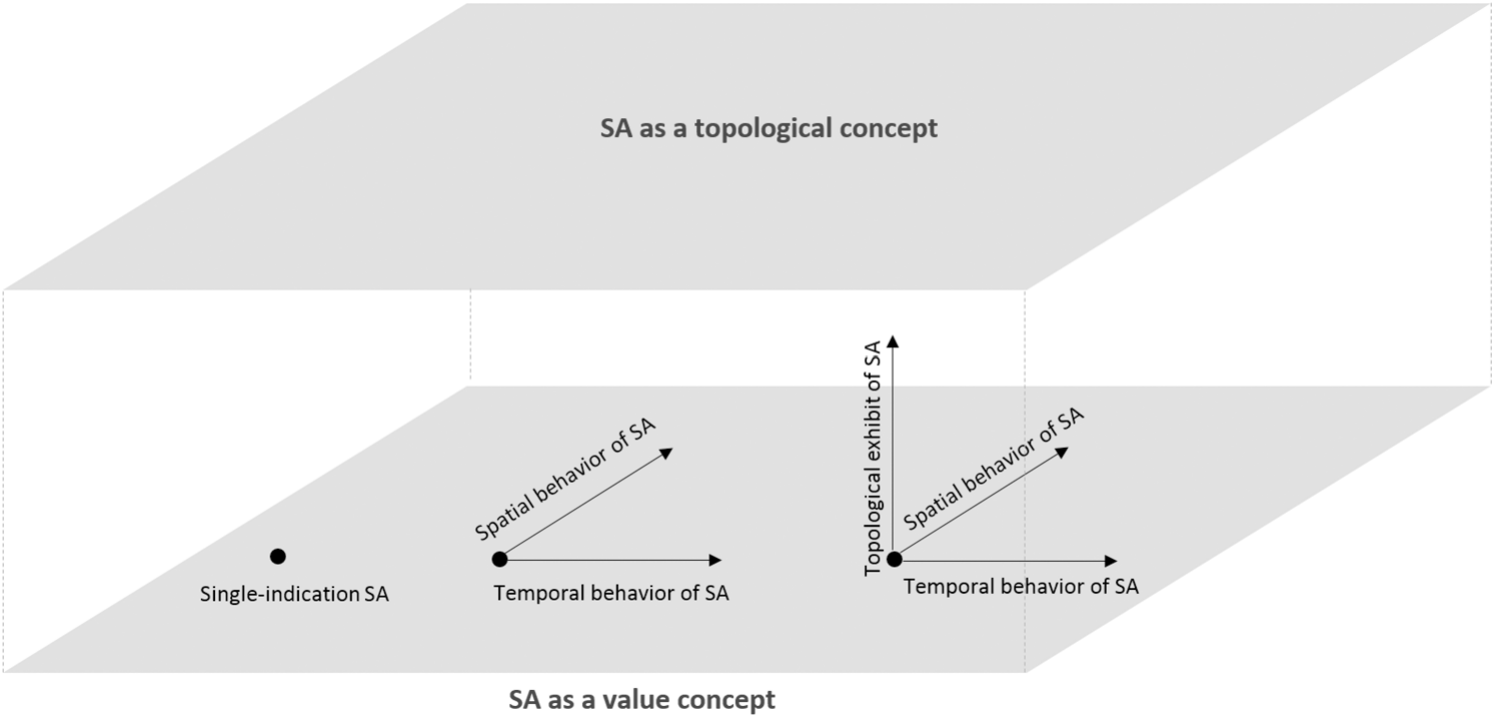
Evolution of SA methodology.

Recent studies demonstrate how SA can be applied to evaluate the temporal [2, 23] and spatial [24–26] complexities of models. Temporal SA explores the regions in the time series of sensitivities where a particular input dominates the others. Spatial SA investigates the spatial heterogeneities of models, especially in the geographic space [24]. Compared to the traditional SA, in which only the final model sensitivities are of interest, temporal SA and spatial SA examine the behaviors of a model over time and space. SA has been therefore extended from a scalar (single-indication) type of analysis to a time series or a layer that contains spatially differentiated information (Fig 11). Following the previous works, TOSA adds a new dimension to comprehensive model evaluation, where SA is applied as a topological concept, other than a value concept (Fig 11). As a consequence, a new school of SA methods may emerge, promoting our understanding of models and the modeled systems. We hypothesize that TOSA may be especially useful in model-based scenario analysis, contributing to more solid understanding of factors that are critical in identifying similar output scenarios.

The proposed methodology is obviously in its infancy. Future work will focus on three aspects of TOSA development. First, using a number of case studies, we plan to compare and contrast TOSA with the most common value-based approaches (most notably, variance-based SA). Second, we will identify conditions in which the use the simpler centroid-based TOSA metrics is insufficient. Third, the temporal complexity of TOSA could be as high as O(*m*!) times O(*n* ^*2*^). In the case study, the longest experiment took nearly nine hours to finish (on a dual processor desktop computer with 32 GB RAM, running Windows 7). We argue, however, that modelers should be first concerned with getting the right answer, and then focus on reducing the processing time of the evaluation procedure. We do not expect TOSA to be done in real time. In many applications, post-modeling analyses are performed to obtain more knowledge about the studied systems, rather than assisting in real time decision making. Even when the real time analysis is required, some technologies may be leveraged to expedite the calculation, such as cloud computing. In the future, we plan to optimize the execution to provide a new tool that can be applied to diverse complex modeling applications.

## Conclusions

SA is undoubtedly a critical component in shaping our understanding of modeled systems. Current SA approaches quantify sensitivities on the basis of change in values. This paper proposes a different approach to evaluating model behavior—an approach based on topology that represents the connectivity of multidimensional data points. Datasets with identical statistical features (e.g., variance) may be differently spaced, resulting in diverse topological structures. As a result, two factors of identical influence on output variability (measured using the common SA approaches), may have a different effect when evaluated within the topological space.

The proposed Topology-Oriented Sensitivity Analysis captures the topological difference between the pre-model data space and post-model data space. It defines sensitivities of a particular variable as the contributed marginal and interactive topological changes when this variable is added to the model. When the data space demonstrates more volatility after a variable is added, it suggests a high level of model sensitivity to this variable. Measuring volatility of data space is not a trivial task, since the dimensionality of data space keeps changing during TOSA calculation (removing/adding dimensions). Therefore, as a benchmark, we calculate the ratio of the output space topology to the input space topology.

Topology-based sensitivity analysis introduces an alternative way of looking at a model and its data. It introduces new opportunities for investigating the hidden but potentially critical characteristics of modeled systems. More efforts are therefore urged to extend this paradigm of model evaluation.

## Supporting Information

S1 Text VBA code for Distance based TOSI. (TXT)

S2 Text VBA code for Centroid based TOSI. (TXT)

S3 Text VBA code for Vector based TOSI. (TXT)

S4 Text VBA code for Centralized Vector based TOSI. (TXT)

S5 Text SAS code for Distance based TOSI. (TXT)

S6 Text SAS code for Centroid based TOSI. (TXT)

S7 Text SAS code for Vector based TOSI. (TXT)

S8 Text SAS code for Centralized Vector based TOSI. (TXT)

S9 Text R code for all four TOSI’s. (TXT)
